# The production of enantiomerically pure 1-phenylethanol by enzymatic kinetic resolution method using response surface methodology

**DOI:** 10.3906/kim-1912-23

**Published:** 2020-10-26

**Authors:** Ayşe BOZAN, Rahime SONGÜR, Ülkü MEHMETOĞLU

**Affiliations:** 1 Department of Chemical Engineering, Faculty of Engineering, Ankara University, Ankara Turkey

**Keywords:** 1-phenylethanol, enzymatic kinetic resolution, chiral alcohol, Novozyme 435, response surface methodology

## Abstract

As the enantiomers of 1-phenylethanol are valuable intermediates in several industries, the lipase catalyzed kinetic resolution of (R,S) -1-phenylethanol is a relevant research topic. In this study, the goal was to determine the optimum reaction parameters to produce enantiomerically pure 1-phenylethanol by lipase (Novozyme 435) catalyzed kinetic resolution using response surface methodology (RSM). Reactions were performed with 40–400 mM (R,S)-1-phenylethanol, 120–1200 mM vinyl acetate and 2–22 mg/mL biocatalyst concentrations (BC _L_ ), at 20–60 °C and with a stirring rate of 50–400 rpm for 5–120 min. The samples were analyzed using high performance liquid chromatography (HPLC) with a Chiralcel OB column. Optimum reaction parameters to reach 100% enantiomeric excess for the substrate ( *ee*_*s*_ ) were determined as follows: substrate concentration (C _s_ ): 240 mM, BC _L_ : 11 mg/mL, at 42 °C with a reaction time of 75 min. Model validation was performed using these conditions and *ee*_*s*_ was calculated as 100%, which indicates the predicted model was efficient and accurate. When compared to the literature, it was observed that the reaction time decreased significantly. This is an important result considering the industrial scale perspective.

## 1. Introduction

Enantiopure chiral compounds are crucial intermediates in several industries, such as the pharmaceutical, agricultural, fine chemicals, and food industries [1]. In particular, in the pharmaceutical industry, the importance of chirality and enantiopure chiral compounds was realized when the unfortunate outcomes of racemic drugs were revealed. As seen in the example of thalidomide, in some cases, the effects of a distomer might result in irreparable damage [2]. This unwanted outcome showed the strong association between chirality and biological activity. In this way, enantiopure drug intermediates and seeking chiral replacements dominated the pharmaceutical industry so much that the share of chiral drugs in the pharmaceutical drug industry has been growing continuously ever since, especially over the last two decades [1, 3–6]. In fact, 80% of the drugs developed during this time were chiral molecules [5].

Enantiomerically pure compounds are obtained from chiral pool components by synthesis, racemic mixtures by resolution, and prochiral substrates by asymmetric synthesis [4]. Of all these methods; enzymatic kinetic resolution, in which the two enantiomers of a racemic mixture react at different rates with a chiral entity, is mostly preferred considering the high activities, selectivities, and mild operating conditions and is proven to be more economical than asymmetric synthesis [1,3]. In order to ensure high selectivity in the enzymatic kinetic resolution, the difference between the reactions rates of each enantiomer should be as large as possible [7]. Once this prerequisite is satisfied, enzymatic kinetic resolution is a promising and attractive method due to its properties, such as, being environmentally friendly, having strong specificity under mild operation conditions, and ease in separation [8].

These days, the effects of biocatalysts are gaining importance in biotechnology [9]. To ensure green chemistry applications, enzymes or whole cells are being used instead of chemical catalysis [10]. According to reviews, it has been revealed that more than 13,000 enzyme catalyzed reactions have been performed at a laboratory scale [11]. Considering their significant stereo-, regio-, and chemo-selectivities, enzymes undertake an attractive and promising role in the synthesis of chiral molecules [7,12].

Lipases (triacylglycerol acyl hydrolases, EC 3.1.1.3) are used as biocatalysts in various reactions both in aqueous and nonaqueous media. They are widely distributed in nature and can be found in several sources such as animals, plants, bacteria, yeast and fungi [13,14].

The outstanding nature of the lipases for accepting abundant substrates, preserving activity in organic solvents, enantioselectivity, efficiency even in mild reaction conditions, stability, lack of a cofactor requirement, easy availability, and relatively reasonable prices make them the most attractive candidate to catalyze chiral reactions [1,6,9–10, and 14,15]. When used in an immobilized form, lipases stand out as a useful tool for industrial scale production and is used in several fields such as detergent, food, animal feed, paper and cellulose, cosmetics, chemical synthesis, and pharmaceutical industries [1,5,14].

Novozyme 435 is one of the most preferred lipases, having an activity of more than 5,000 PLU/g (propyl laurate units/g). It is *Candida antarctica* Lipase B (CALB) that is commercially immobilized onto macroporous acrylic polymer resin (Lewatit VP OC 1600) [16].

1-phenylethanol is a secondary alcohol. Chiral phenylethanol derivatives are used as chiral building blocks and intermediates in several industries such as pharmaceuticals, agrochemicals, flavours, and fine chemicals. For that reason, their demand is increasing [17,18]. (R)-1-phenylethanol is especially used as a fragrance, solvatochromic dye, ophthalmic preservative, and as an inhibitor of cholesterol intestinal adsorption in pharmaceutical, cosmetic, and chemical industries [17]. The esterified derivatives of (R,S)-1-phenylethanol also have many applications in perfumeries, soaps, detergents, cosmetics, room sprays, deodorants, and flavors [18].

Considering the importance of chiral 1-phenylethanol in several industries, the lipase catalyzed kinetic resolution of (R,S)-1-phenylethanol has been investigated by several research groups [3, 19–27].

In case of transesterification, parameters such as the lipase type and loading, reaction temperature, acyl donor type, molar ratio of acyl donor to substrate ((Ac:S) _MR_ ), substrate concentration, as well as solvent type and characteristics have been investigated [3, 12, 17–18, 26–27].

According to several papers in the field, Novozyme 435 as the biocatalyst, vinyl acetate as the acylating agent [3,20], and hexane as the reaction medium [12,18] gave better results in terms of yield, conversion, and enantioselectivities.

Dhake et al. [12] applied steapsin lipase (having an activity of more than 20 U/mg) for the kinetic resolution of (R,S)-1-phenylethanol and its derivatives using vinyl acetate as the acyl donor and hexane as the reaction solvent. In their study, the optimum reaction conditions were as follows. The substrate concentration: 166 mM, molar ratio of acyl donor to substrate 4:1, reaction temperature: 55 °C, reaction time: 24 h and lipase amount: 23.3 mg/mL. Under these conditions, the conversion, the enantiomeric excess for the substrate ( *ee*_*s*_ ), the enantiomeric excess for the product ( *ee* *p* ), and the enantiomeric ratio (E) were calculated as 48%, 85%, 92% and 66, respectively. In another study, Kamble et al. [18] used cutinase (from the newly isolated fungal strain *Fusarium sp. ICT SAC1* , having 2.12 U/mg activity) as the biocatalyst, vinyl acetate as the acyl donor and hexane as the reaction solvent. Reactions were performed at 200 mM substrate concentration and 13.3 mg/mL cutinase concentration at 40 °C, in a period of 18 h. Under these conditions, the conversion and *ee*_*s*_ were calculated as 44.21%, and 79.23%, respectively. In these studies, the type of lipase was different than this study.

Among the papers published on this subject, very few researchers applied statistical techniques together with experimental results to optimize the process parameters. Li et al. [17] applied the kinetic resolution of (R,S)-1-phenylethanol by using a different lipase ( *B. cenocepacia* ) produced by *B. cepacia* G63 strain and reaction parameters were optimized by response surface methodology (RSM). In their work, the optimized conditions were as follows. The molar ratio of acyl donor to substrate is 4.7:1, the reaction temperature: 53.4 °C, the reaction time: 18.6 h, the biocatalyst loading: 20 mg/mL (activity was 1392.2 U/min/g protein), and the substrate concentration: 200 mM. Under these conditions, *ee*_*s*_ was 99.22%. When compared to our study, the type of lipase ( *B. cenocepacia* ) and the reaction medium (heptane) were different.

In the earlier days of experimental optimization, the main idea was to change one variable at a time by keeping the rest of the variables’ constant. However, in this way the interactive effects between the variables could not be determined. Also, the number of experiments had to be increased which meant wasted time, utility, and raw material [28]. In order to overcome this problem RSM has been applied. When using this method, statistical and mathematical techniques are used simultaneously in order to develop, improve, and optimize processes [29].

In this study, the Novozyme 435 catalyzed enzymatic kinetic resolution of (R,S)-1-phenylethanol was performed and the optimum reaction parameters were determined through RSM using central composite design (CCD). Selected independent variables for RSM were the substrate concentration, the temperature, the reaction time, and the biocatalyst loading. The aim of this study was decreasing the reaction time and the biocatalyst consumption when compared with the literature results as it is a necessity for industrial scale production.

## 2. Materials and methods

### 2.1. Materials

(R,S)-1-phenylethanol (CAS No. 98-85-1), (R)- and (S)-1-phenylethanol (CAS No.1517-69-7 and 1445-91-6) , 1-phenylethyl acetate (CAS No. 93-92-5), vinyl acetate, hexane and methyl tert butyl ether (MTBE) were purchased from Sigma-Aldrich (Sigma-Aldrich Corp., St. Louis, MO, USA) and Merck KGAA, Darmstadt, Germany. The commercial lipase Novozyme 435 (activity more than 5,000 PLU/g) was kindly donated by Novozymes.

### 2.2. Lipase catalyzed kinetic resolution of (R-S)-1-phenylethanol

40–400 mM of (R,S)-1-phenylethanol dissolved in n-hexane was added to a 25 mL sealed glass bioreactor. 120–1200 mM of vinyl acetate and a 2–22 mg/mL biocatalyst were added to the reaction mixture. The total reaction volume was 5 mL. The reactions were performed for 5–120 min, at 20–60 °C with a stirring rate of 50–400 rpm. All the experiments were carried out in duplicate at least and the mean values were reported. At the end of the reaction time the biocatalyst was removed by filtration and the solvent was evaporated under vacuum. The residual was dissolved in MTBE and filtered through a 0.45 mm filter. The samples were analyzed using high performance liquid chromatography (HPLC), thermo finnigan spectra system.

### 2.3. HPLC analysis

The concentrations of the enantiomers of (R,S)-1-phenylethanol were determined by HPLC with a Chiralcel OB column (Daicel Chemical, Daicel Corporation, Japan). The conditions of the HPLC analysis for the enantiomers are given in Table 1. The *ee*_*s*_ % was calculated using Eq (1) [17,18,21, 30], where R and S stand for the concentrations of the (S)- and (R)-enantiomers of 1-phenylethanol after the reaction.

(1)ees%=[(R-S)/(R+S)]x100

**Table 1:** The conditions of the HPLC analysis.

Column	Chiralcel OB (4.6x250 mm)
Mobile phase	n-hexane:isopropyl alcohol (95 : 5) (v / v)
Flow rate	0.9 mL/min
Injection volume	10 μL
Column temperature	30 °C
UV detector (wavelength)	254 nm
Analysis duration	30 min
Retention times	S-1-phenylethanol : 8 min R-1-phenylethanol : 11 min

## 3. Results and discussion

**Figure 1:**
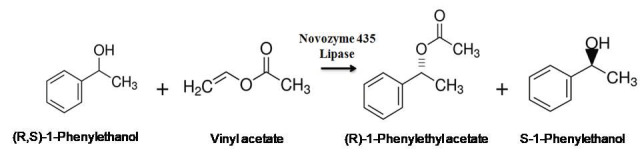
Enzymatic kinetic resolution of (R,S)-1-phenylethanol.

The enantioselective transesterification reaction catalyzed by Novozyme 435 is shown in Figure 1.

In this study, the reaction was performed using the Novozyme 435 lipase. Based on the results of the previous papers in the field; vinyl acetate [3,20] was preferred as the acyl donor in order to achieve an irreversible reaction and hexane [12,18] was preferred as the reaction solvent due to its nonpolar nature and simple molecular structure which effect the enzyme activity and *ee*_*s*_ positively. In all the experiments, only (R)-1-phenylethanol was reactive. This result is in agreement with the literature [3,24,27].

In the preliminary experiments, the effects of the reaction temperature and time, the substrate concentration, the biocatalyst loading, and the stirring rate on *ee*_*s*_ were investigated in order to determine the predominant independent variables and their intervals for RSM.

### 3.1. Preliminary experiments in order to determine the independent variables for RSM

**Figure 2:**
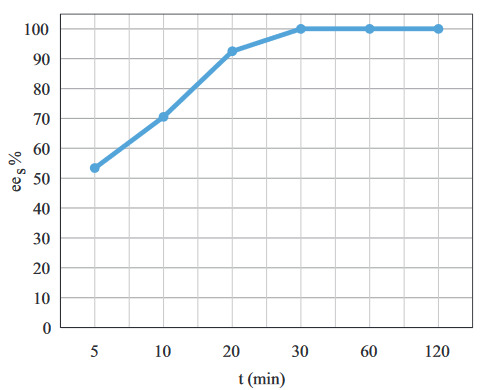
The effect of reaction time on *ee _s_* (C _s_ = 120 mM, (Ac : S) _MR_ = 3: 1, BC _L_ = 20 mg/mL, T = 25 °C, stirring rate = 250 rpm).

Initially, the effect of the reaction time on *ee*_*s*_ was investigated. As seen in Figure 2, *ee*_*s*_ increased with the reaction time. The value of *ee*_*s*_ reached 100% after 30 min with a 20 mg/mL biocatalyst loading. When compared with the results in the literature, it was observed that *ee*_*s*_ became 100% in a shorter reaction time in this study [3,12,17,18,20,24,27]. This may be attributed to higher biocatalyst loading, the high activity of Novozyme 435, and also the nature of the solvent.

According to the literature, the significantly higher activity of Novozyme 435 when compared with the other lipases comes from the characteristic property of *Candida antarctica* lipase B, which has a very small and simple lid at its active center [31].

Additionally, in this study, the reaction time (30 min) to reach 100% *ee*_*s*_ was much shorter than the literature results in which Novozyme 435 was also used as the lipase. This could be attributed to the selection of the reaction medium. For example, Habulin and Knez [3] reported that the reaction time to reach 99% *ee*_*s*_ was 3 h (at 40 °C, 500 rpm using vinyl acetate as acyl donor and [Bmim][PF6] as solvent). In comparison to their research, the reaction time was shorter in our study. Kamble et al. [18] explained that the selection of the solvent is critical in order to retain structural conformity and enzyme activity. According to their work, hexane was the best solvent. This resulted from the nonpolar nature of hexane which does not strip out the tightly bound essential water layer from the enzyme surface and thereby the activity of the enzyme is preserved. According to the literature, it has been stated that the molecular structure of the solvent also plays an important role in the enzyme activity and *ee*_*s*_ . Short carbon chain alkanes were advantageous when compared with the organic solvents having other functional groups [17,18]. Taking this into consideration, it is understood that a suitable solvent was selected for this reaction.

In addition to the type of biocatalyst and the reaction medium, biocatalyst loading is also one of the most important parameters during optimization studies, because high biocatalyst loading increases the rate. However, at the same time, it increases the cost of the reaction. In this study, the effect of biocatalyst loading was investigated between 2–20 mg/mL.

**Figure 3:**
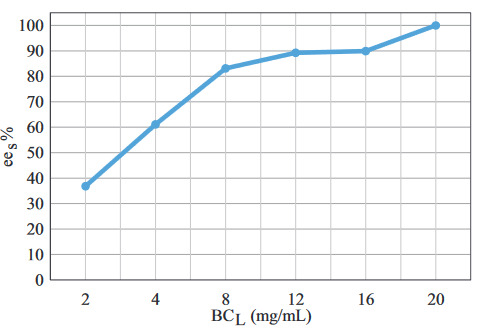
The effect of biocatalyst loading on *ee _s_* (C _s_ = 120 mM, (Ac : S) _MR_ = 3:1, t = 30 min, T = 25 °C, stirring rate = 250 rpm).

In response to the increase in biocatalyst loading, *ee*_*s*_ reached 100% at 20 mg/mL (Figure 3). This may result from the increase in the number of enzyme active sites interacting with the substrate. A total of 20 mg/mL biocatalyst loading was selected for further experiments.

**Figure 4:**
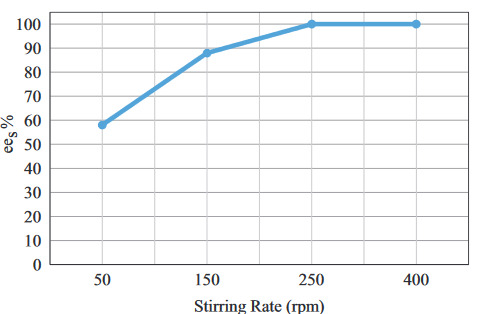
The effect of stirring rate on *ee _s_* (C _s_ = 120 mM, (Ac : S) _MR_ = 3:1, t = 30 min, BC _L_ = 20 mg/mL, T = 25 °C).

Novozyme 435 is an immobilized catalyst with a porous structure. Initially, reactant molecules must diffuse from the bulk solution to the internal surface in order to interact with the lipase. When the optimum stirring rate is reached in the reaction medium, the thickness of the solvent film around the catalyst particle diminishes and the effect of the external mass transfer limitation becomes negligible [32]. For this reason, the effect of the stirring rate on *ee*_*s*_ was also investigated. External mass transfer limitations were eliminated at 250 rpm and therefore this rate was used for further experiments (Figure 4).

**Figure 5:**
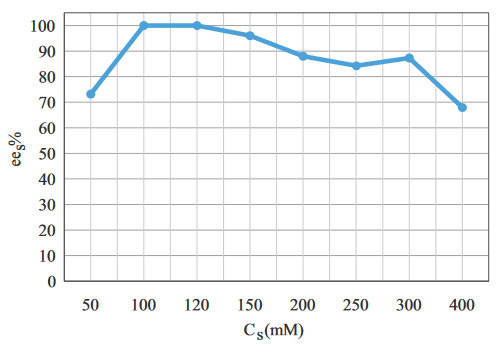
The effect of Cs on *ee _s_* ( t = 30 min, (Ac : S) _MR_ = 3:1, BC _L_ = 20 mg/mL, T = 25 °C, stirring rate = 250 rpm).

The effect of substrate concentration on *ee*_*s*_ is shown in Figure 5. After 120 mM, a decrease in *ee*_*s*_ was observed. This phenomenon indicated the lack of acyl-enzyme complex for the substrate. Therefore, 120 mM was selected for further experiments.

**Figure 6:**
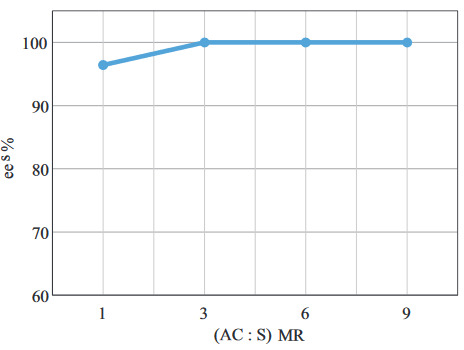
The effect of (Ac : S) _MR_ on *ee _s_* (C _s_ = 120 mM, t = 30 min, BC _L_ = 20 mg/mL, T = 25 °C, stirring rate = 250 rpm).

The effect of the molar ratio of the acyl donor to the substrate on *ee*_*s*_ is shown in Figure 6. As seen, an increase in *ee*_*s*_ was observed at a molar ratio up to 3:1. This ratio was also supported by several papers as it ensures the irreversibility of the reaction. In this case, the nature of the acyl donor (vinyl acetate) also guaranteed the irreversibility of the reaction by resulting in vinyl alcohol (which immediately turns into acetaldehyde and renders the reaction irreversible) [4,10, 27]. A further increase beyond 3:1 did not affect the *ee*_*s*_ and therefore this ratio was applied for the following experiments.

**Figure 7:**
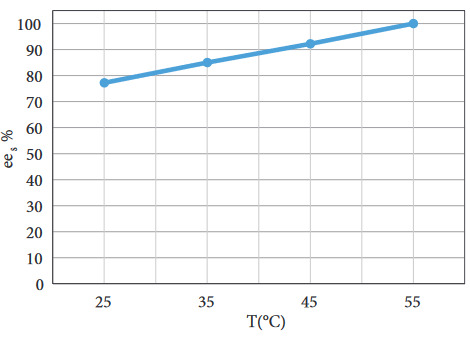
The effect of T on *ee _s_* (C _s_ = 120 mM, (Ac : S) _MR_ = 3 : 1, BC _L_ = 20 mg/mL, stirring rate = 250 rpm, t = 15 min).

Enzymatic reactions are sensitive to temperature. But immobilized lipase enables higher reaction temperatures by improving the stability of the enzyme. The optimum reaction temperature range for Novozyme 435 was determined to be between 30–60 °C according to the product catalogue. The effect of the temperature was investigated between 25 °C to 55 °C during 15 min reaction time (Figure 7).

The effect of the temperature was found to be significant. When the reaction temperature was increased from 25 °C to 55 °C, the *ee*_*s*_ increased from 77% to 100%. This increase could be attributed to the increased energetic collisions between the enzyme and substrate molecules. These collisions promote the formation of the enzyme-substrate complex by favoring a faster reaction [18].

### 3.2. The application of RSM

The independent variables for RSM were selected according to the results of the preliminary experiments. From these results it has been understood that, the effects of the stirring rate and the molar ratio of acyl donor to substrate were minor in respect to the other parameters. In line with the main aim of RSM, only the variables which seem to have “major effects” on the reaction were selected as independent variables. These independent variables were substrate concentration, temperature, reaction time, and biocatalyst loading. The levels of the independent variables were also determined according to the preliminary experiments and shown in Table 2. For all the experiments, the molar ratio of the acyl donor to the substrate was 3:1 and the stirring rate was 250 rpm.

CCD is a statistically-based experimental design approach and it is commonly used in order to estimate second order response surfaces [33]. CCD was applied for the above independent variables and the number of the experiments (30 in total) were calculated according to N = k ^2^ +2k+cp. In this formula, k is the factor number and cp is the replicate number of the central point [28]. The experimental design was carried out in Design Expert 7.0.0 (Stat-Ease, Inc., Minneapolis, MN, USA). Experimental conditions and the values of the response are shown in Table 3.

The following second-order polynomial model (Eq (2)) was applied to obtain the relationship between the response (Y), in this case *ee*_*s*_ and the experimental parameters in coded values.

Y = 100 − 2.73A + 6.82B + 3.16C + 8.05D + 4.56AB + 1.93AC + 3.56AD − 2.0BC − 3.64BD − 2.5CD − 0.86A2 − 5.28B2 − 0.17C2 − 3.92D2 (2).

In this equation, A, B, C, and D are the coded values of the substrate concentration, the biocatalyst loading, the temperature, and the reaction time, respectively. The model is in accordance with the quadratic model.

The results of the second-order response surface model fitting the form of ANOVA (analysis of variance) are given in Table 4.

**Table 2:** Coded and real values of the independent variables in RSM.

Factor	Variable	Unit	–2	–1	0	1	2
A	C _s_	mM	40	130	220	310	400
B	BC _L_	mg/mL	2	7	12	17	22
C	T	°C	20	30	40	50	60
D	t	min	10	35	60	85	110

**Table 3:** Experimental conditions and the real value of the response.Experiment no.A(Cs (mM))B (BCL)(mg/mL))C (T(°C))D (t(min))(Real value of the response).

Experiment no.	A(C _s_ (mM))	B (BC _L_ )(mg/mL))	C (T(°C))	D (t(min))	(Real value of the response) ( *ee _s_* %)
1	220	12	40	60	100.00
2	130	17	30	35	94.00
3	130	17	50	85	100.00
4	310	17	30	85	100.00
5	220	12	40	60	100.00
6	220	12	40	60	100.00
7	310	17	50	85	100.00
8	220	2	40	60	48.80
9	220	12	40	60	100.00
10	220	12	40	10	56.70
11	220	22	40	60	97.00
12	310	7	50	35	74.50
13	130	17	50	35	92.20
14	310	17	30	35	89.60
15	310	7	50	85	100.00
16	220	12	20	60	86.70
17	130	7	30	35	89.00
18	130	17	30	85	100.00
19	220	12	60	60	100.00
20	220	12	40	60	100.00
21	310	7	30	85	95.40
22	130	7	30	85	100.00
23	400	12	40	60	90.80
24	310	7	30	35	49.50
25	130	7	50	85	100.00
26	130	7	50	35	100.00
27	40	12	40	60	90.40
28	310	17	50	35	100.00
29	220	12	40	110	100.00
30	220	12	40	60	100.00

**Table 4:** ANOVA results for the model.

Source	Sum of squares	Degree of freedom (d _f_ )	Mean square	F value	P value Prob > F	
Model	5135.68	14	366.83	4.35	0.0038	Significant
A - C _s_	178.22	1	178.22	2.12	0.1664	
B - BC _L_	1117.93	1	1117.93	13.27	0.0024	
C - T	239.40	1	239.40	2.84	0.1125	
D - t	1555.26	1	1555.26	18.46	0.0006	
AB	333.06	1	333.06	3.95	0.0653	
AC	59.29	1	59.29	0.70	0.4147	
AD	203.06	1	203.06	2.41	0.1414	
BC	64.00	1	64.00	0.76	0.3972	
BD	211.70	1	211.70	2.51	0.1338	
CD	100.00	1	100.00	1.19	0.2931	
A ^2^	20.11	1	20.11	0.24	0.6322	
B ^2^	765.03	1	765.03	9.08	0.0087	
C ^2^	0.78	1	0.78	9.272E-003	0.9246	
D ^2^	421.21	1	421.21	5.00	0.0410	
Residual	1263.63	15	84.24			
Lack of fit	1263.63	10	126.36			
Pure error	0.000	5	0.000			
Cor total	6399.31	29				

The F value of 4.35 suggests that the model is statistically significant. The adequate precision value of 8.654 is higher than 4. The Fisher F-test with a very low probability value (0.0038) confirms the adequacy of the quadratic model. The P value < 0.05 indicates the significance of the model. In this case, B, D, B ^2^ , and D ^2^ are significant model terms. The most significant term is the reaction time with a Prob > F value of 0.0006. The R ^2^ value is determined to be 0.8025. It is seen from the model that the linear terms B, C, D, as well as the quadratic terms AB, AC, AD have positive effects on *ee*_*s*_ . However, the linear term A, as well as the quadratic terms BC, BD, CD, A ^2^ , B ^2^ , C ^2^ , and D ^2^ have negative effects on *ee*_*s*_ .

**Figure 8:**
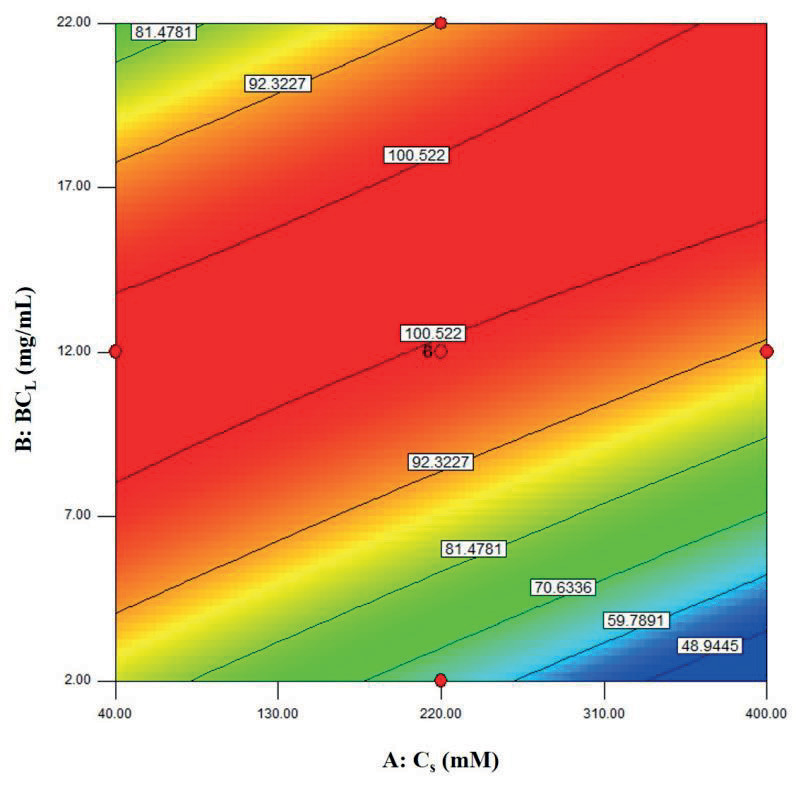
Contour plots showing the dual effect of the independent variables Cs (mM) and BCL (mg/mL) on ees (T = 40 °C, t = 60 min).

**Figure 9:**
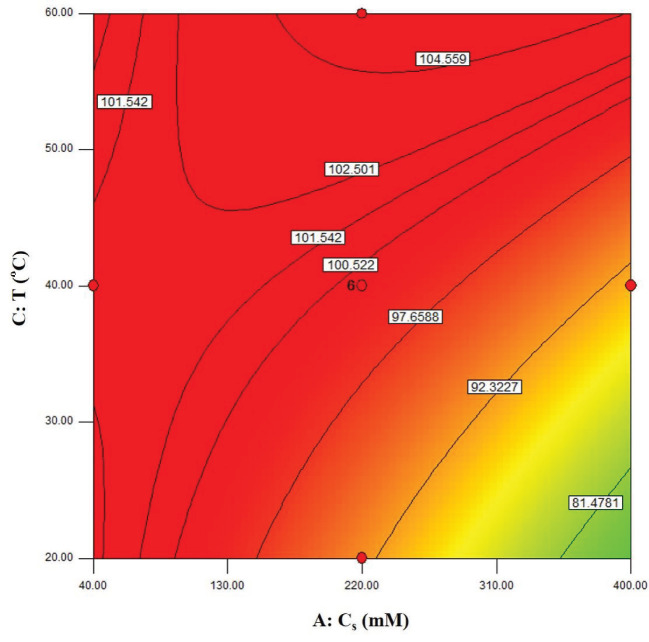
Contour plots showing the dual effect of the independent variables Cs (mM) and T(°C) on ees (BCL = 12 mg/mL, t = 60 min).

**Figure 10:**
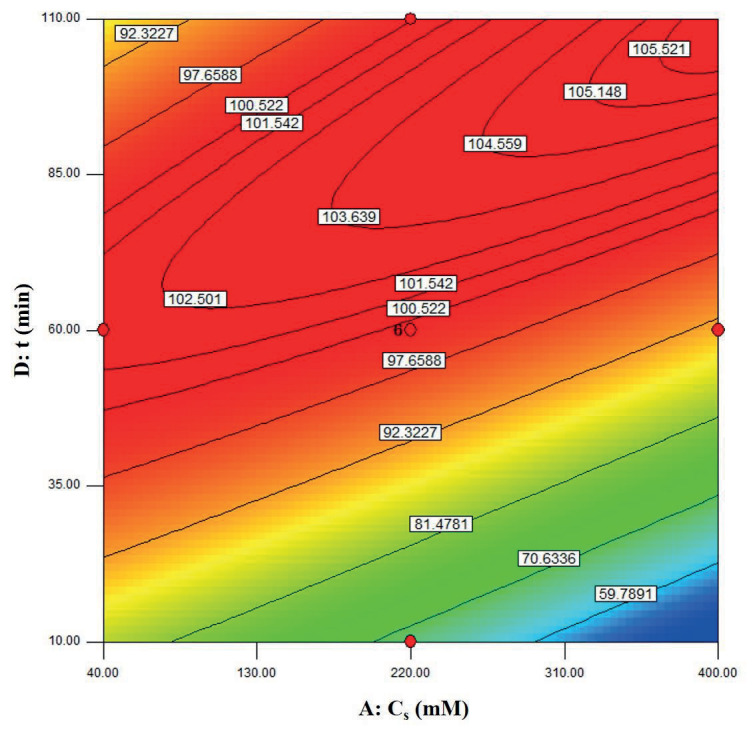
Contour plots showing the dual effect of the independent variables Cs (mM) and t (min) on ees (BCL = 12 mg/mL, T = 40 °C).

**Figure 11:**
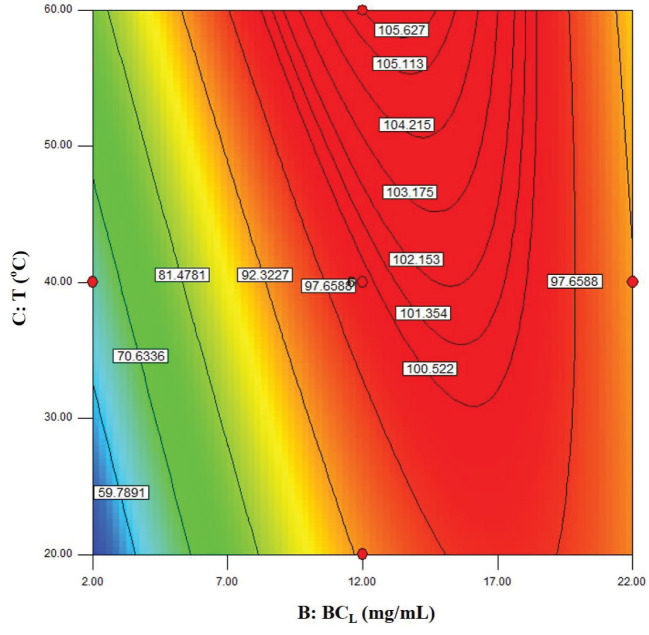
Contour plots showing the dual effect of the independent variables BCL (mg/mL) and T (°C) on ees (Cs = 220 mM, t = 60 min).

**Figure 12:**
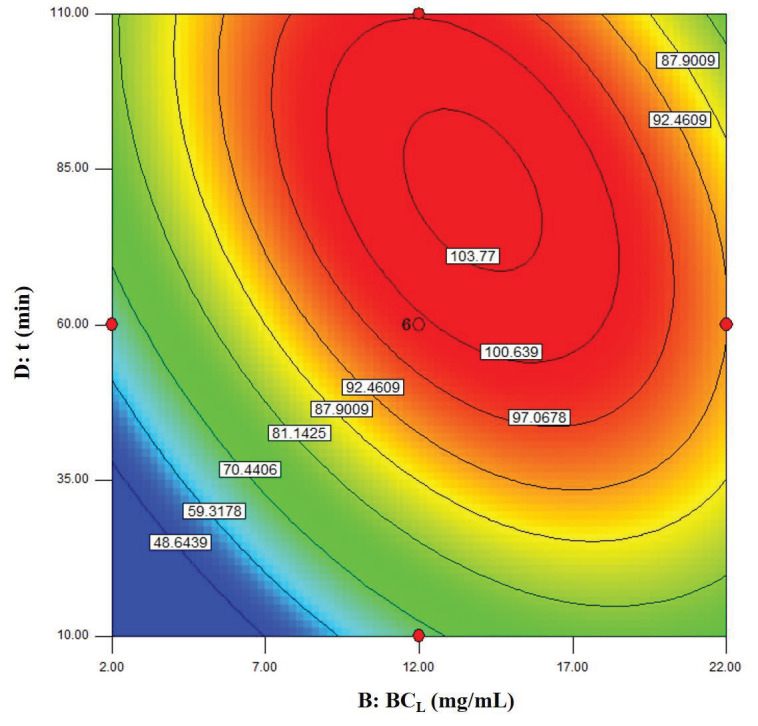
Contour plots showing the dual effect of the independent variables BCL (mg/mL) and t (min) on ees (Cs = 220 μM, T = 40 °C).

**Figure 13:**
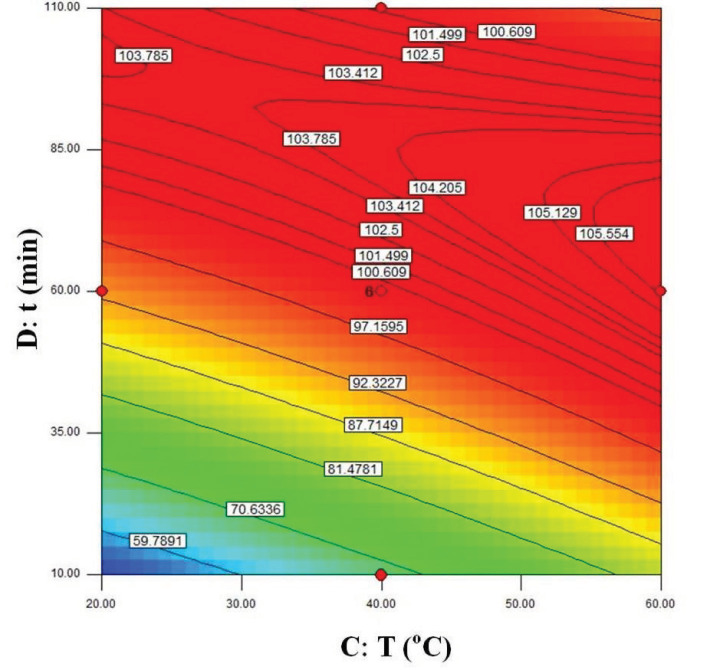
Contour plots showing the dual effect of the independent variables T (°C) and t (min) on ees (Cs = 220 mM, BCL = 12 mg/mL).

The visualization of the predicted model equation can be obtained using surface response plots [28]. The response plots which represent the dual effects of the independent variables on the response are given in Figures 8–13.

According to Figure 8, increasing the substrate concentration at a constant biocatalyst loading, decreased *ee*_*s*_ values. This could be explained by the fact that there could not be enough active sites to interact with the substrate and thus the reaction could not be completed. On the other hand, when the biocatalyst loading was quite high, lipase particles adhered to the walls of the reactor in bulk. This diminished the mass transfer rate and decreased the *ee*_*s*_ value. This was also experienced by Kamble et al. [18].

In Figure 9, the relation between the substrate concentration and the reaction temperature is shown. According to the figure, a saddle point was observed which refers to an inflexion point between a relative maximum and a relative minimum [28]. If the contour plot is in the form of a saddle, this means that the interactions between the variables are significant [34]. The effect of the temperature was predominant at higher substrate concentrations. When the reaction temperature increased, this caused an increase in the amount of the enzyme-substrate complex, and faster reaction rates [18].

In Figure 10, the relation between the substrate concentration and the reaction time is shown. According to the figure, the effect of time was predominant at a higher substrate concentration. On the other hand, at a lower substrate concentration (as the reaction completed even at shorter time) the increase in reaction time did not affect the *ee*_*s*_ value significantly.

The relation between biocatalyst loading and the temperature is shown in Figure 11. The effect of the temperature was predominant at lower biocatalyst loading.

In Figure 12, the relation between biocatalyst loading and the reaction time is shown. It has been understood that the maximum *ee*_*s*_ value is located inside the experimental region. As seen, there was a significant relation between biocatalyst loading and reaction time. It has been also understood that the effect of the reaction time was more significant at lower biocatalyst loadings.

In Figure 13, the relation between the reaction temperature and the time is shown. A saddle point was observed which indicates that there was a significant interaction between temperature and time. After 60 min, even at room temperature, high *ee*_*s*_ values were reached. As the temperature stability of the immobilized lipase is high, no loss in lipase activity was observed even at 60 °C.

In order to determine the value of the independent variables which maximize the *ee*_*s*_ value, the model equation was solved and the reaction conditions that makes *ee*_*s*_ at 100% were given as follows. The substrate concentration : 240 mM, biocatalyst loading : 11 mg/mL, reaction temperature : 42 °C, and reaction time : 75 min.

In order to validate the model an experiment was performed at the optimum reaction conditions and *ee*_*s*_ was calculated to be 100%. This indicated that the predicted model was efficient and accurate.

Compared to previous studies in this field, the shortest reaction time (75 min) was achieved in this specific study, which was the completion of the reaction at 240 mM. This time is remarkable considering the literature results range from 2 to 24 h at a substrate concentration ranging from 5 mM to 1 M [3,12,17,18,20,21,23,24,26,27].

When compared to the literature results (in which different biocatalysts such as steapsin lipase, *B. cenocepacia* lipase, and cutinase were used), the reaction time decreased from 18–24 h to 75 min in this study at a similar substrate concentration (ranging between 160 and 200 mM) [12,17,18]. This improvement can be attributed to the catalytic performance of the Novozyme 435 lipase.

Additionally, better results were reached when compared with the previous investigations in which Novozyme 435 lipase was also used [3,27]. In these studies, the time for the completion of the reaction was higher (3–5 h). Kirilin et al. [27] reported that the time to complete the reaction was 5 h even though a lower substrate concentration (20 mM) was applied. According to the authors, this progress can be attributed to the well-chosen solvent type and the efficient optimization of the reaction parameters with the contribution of RSM.

By using RSM, the reaction parameters were optimized efficiently with the contribution of the statistical methods and also by considering the interactive effects between the parameters. When the optimum conditions reached in RSM were compared with the results of the preliminary experiments, it has been observed that the substrate concentration doubled even when the lipase concentration decreased by almost 50%. This improvement is advantageous in terms of the operational costs. Even though there is also increase in reaction time and temperature in RSM, the authors believe that decreasing the costs (by increasing the substrate concentration and decreasing the lipase concentration) is more preferable in terms of operational feasibility. Thus, optimum RSM conditions are preferable in respect to the results of the preliminary experiments.

In order to scale-up the laboratory experiments, the reusability of the biocatalyst possesses great importance [32]. To determine the reusability of the lipase, consecutive experiments were performed at the optimum reaction conditions. In these experiments, the lipase was filtered after the completion of the reaction, washed with hexane, dried at room temperature, and used for the next experiment.

**Figure 14:**
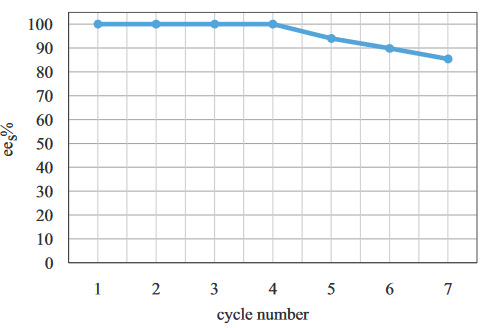
Recycle use of the biocatalyst at optimum reaction conditions (Cs = 240 mM, BCL = 11 mg/mL, T = 42 °C, t = 75 min).

According to Figure 14, it has been determined that the reusability of the biocatalyst is high. No decrease in *ee*_*s*_ was observed up until the 4th cycle. A decrease of approximately 15% was observed in *ee*_*s*_ after the 7th cycle.

## 4. Conclusion

1-phenylethanol is an outstanding chiral secondary alcohol and an important precursor in the synthesis of pharmaceutical, agrochemical, and natural products. In this study, the lipase catalyzed enzymatic kinetic resolution of (R,S)-1-phenylethanol was performed by a transesterification reaction. Hexane, vinyl acetate, and Novozyme 435 were used as the solvent, acyl donor, and biocatalyst, respectively. The optimum reaction parameters to reach 100% *ee*_*s*_ were determined by RSM as follows. The substrate concentration: 240 mM, biocatalyst loading: 11 mg/mL, the reaction temperature: 42 °C and the reaction time: 75 min. For the validation of the model, an experiment was performed at optimum reaction conditions and *ee*_*s*_ was calculated to be 100%. This indicated that the predicted model was efficient and accurate. When compared with the literature results, a significant decrease in reaction time was observed at similar substrate concentrations. This situation might be attributed to the extraordinary catalytic performance of the Novozyme 435 lipase towards secondary alcohols. It has also been observed that the reaction time was lower, even for the studies in which also Novozyme 435 was used as the lipase. This could be explained by the well-chosen reaction medium and the efficient optimization of the reaction parameters with the contribution of RSM.

Considering the importance of the shorter reaction time in regard to the economic aspects for application on an industrial scale, it is believed that this study has made an important contribution to other studies within the field. Reusability studies have also revealed that the biocatalyst could be used up until the 4th cycle without losing its catalytic activity. This result supports the idea that this method could be used beyond the laboratory scale. Approximately 15% decrease was observed in *ee*_*s*_ after the 7th cycle.
